# ATP-consuming futile cycles as energy dissipating mechanisms to counteract obesity

**DOI:** 10.1007/s11154-021-09690-w

**Published:** 2021-11-06

**Authors:** Alexandra J. Brownstein, Michaela Veliova, Rebeca Acin-Perez, Marc Liesa, Orian S. Shirihai

**Affiliations:** 1 Metabolism Theme, David Geffen School of Medicine, University of California, Los Angeles, CA 90095 USA; 2 Molecular Cellular Integrative Physiology Interdepartmental Program, University of California, Los Angeles, CA 90095 USA; 3 Department of Molecular and Medical Pharmacology, David Geffen School of Medicine, University of California, Los Angeles, CA 90095 USA; 4 Department of Medicine, Division of Endocrinology, David Geffen School of Medicine, University of California, Los Angeles, CA 90095 USA; 5 Molecular Biology Institute, University of California, Los Angeles, CA 90095 USA

**Keywords:** Brown adipose tissue, Futile cycle, Malate aspartate shuttle, Metabolism, Thermogenesis, Energy expenditure

## Abstract

Obesity results from an imbalance in energy homeostasis, whereby excessive energy intake exceeds caloric expenditure. Energy can be dissipated out of an organism by producing heat (thermogenesis), explaining the long-standing interest in exploiting thermogenic processes to counteract obesity. Mitochondrial uncoupling is a process that expends energy by oxidizing nutrients to produce heat, instead of ATP synthesis. Energy can also be dissipated through mechanisms that do not involve mitochondrial uncoupling. Such mechanisms include futile cycles described as metabolic reactions that consume ATP to produce a product from a substrate but then converting the product back into the original substrate, releasing the energy as heat. Energy dissipation driven by cellular ATP demand can be regulated by adjusting the speed and number of futile cycles. Energy consuming futile cycles that are reviewed here are lipolysis/fatty acid re-esterification cycle, creatine/phosphocreatine cycle, and the SERCA-mediated calcium import and export cycle. Their reliance on ATP emphasizes that mitochondrial oxidative function coupled to ATP synthesis, and not just uncoupling, can play a role in thermogenic energy dissipation. Here, we review ATP consuming futile cycles, the evidence for their function in humans, and their potential employment as a strategy to dissipate energy and counteract obesity.

## Introduction

The prevalence of obesity worldwide has substantially increased, concurrently with the vast array of obesity-associated diseases, including type 2 diabetes, dyslipidemia, hypertension heart disease, and cancer. Obesity develops when calorie intake chronically exceeds total energy expenditure, leading to a rise in the proportion of calories that remain in storage. A therapy for weight loss must, therefore, involve a decrease in calorie intake and/or an increase in energy dissipation out of the organism. While efforts have been put into increasing energy expenditure by increasing exercise, this only worked for a small portion of the population. Similarly, low caloric diet offers only a transient reduction in weight that is followed by weight re-gain [1]. This dynamic nature of weight loss, where a low caloric diet is very successful in the first two weeks, but eventually weight loss stops as the body tends to compensate for the decrease in energy intake, is where the most interesting opportunity lies in [2, 3]. What molecular mechanisms are responsible for blocking weight loss after a couple of weeks of low caloric diet?

For the body to stop losing weight under low caloric diet, energy expenditure must go down. Indeed, recent studies in humans have demonstrated that weight loss by caloric restriction results in a decline in basal metabolic rate (BMR), beyond changes attributed to decreased body weight [1, 4–9]. This adaptive reduction in BMR is a regulated mechanism that limits energy dissipation to conserve tissue mass, mostly by suppressing thermogenesis [10]. This adaptation highlights the ability to preserve energy balance when caloric intake is reduced [1]. The question remains, can we use the same pathways to increase energy dissipation and to counteract obesity? Can defects in the mechanisms regulating energy expenditure be responsible for obesity?

Thermogenesis, which is the production of heat in living organisms, is a regulated process that largely contributes to energy dissipation. Two major processes are responsible for thermoregulatory heat production in mammals – shivering and nonshivering thermogenesis. Shivering, the involuntary contraction of skeletal muscles induced by cold exposure results in heat production that involves ATP-dependent movement. Non-shivering thermogenesis is defined as an increase in metabolic heat production that is not associated with muscle activity and occurs in response to environmental temperature [11]. Non-shivering thermogenesis occurs in several systems, including skeletal muscle and most notably brown adipose tissue [11, 12].

Mitochondrial uncoupling is considered a central component of non-shivering thermogenesis by brown adipose tissue. UCP1, an anion/H^+^ symporter, is an uncoupling protein unique to brown adipose tissue and beige adipocytes. (Table 1) [13–15] UCP1 allows for the re-entry of protons into the mitochondrial matrix bypassing ATP-synthase, thereby uncoupling oxygen consumption from ATP synthesis and dissipating the energy of the proton gradient into heat. [13–15]. Uncoupling is activated in response to decreased environmental temperature (cold-induced thermogenesis) and, with some conflicting evidence, to overfeeding (diet-induced thermogenesis) [16–19]. In addition, UCP1^+^ ‘brown-like’ white adipocytes, also named beige cells, are thermogenically active adipocytes that can emerge in white fat when mice are exposed to cold, as well as in response to catecholamines and other β-adrenergic agonists [18, 20]. Several lines of evidence suggest that beige adipocytes, despite carrying UCP1, also dissipate energy through ATP-consuming processes. [21–24].

**Table 1:** ATP-consuming processes that contribute to energy expenditure. Non-shivering thermogenesis through UCP1-mediated proton gradient dissipation is the main mechanism of BAT-mediated energy expenditure. Additional futile cycles dependent on ATP synthesis and consumption have been demonstrated to contribute to energy dissipation in a UCP1 independent manner in different tissues. UCP1, uncoupling protein 1; AAC, mitochondrial ADP/ATP carrier; SERCA1/2b, sarco/endoplasmic reticulum Ca^2+^-ATPase; RyR, Ryanodine receptor; SLN, Sarcolipin; CK, Creatine Kinase; PEPCK-C, Phosphoenolpyruvate carboxykinase; MPC, mitochondrial pyruvate carrier; LDH, Lactate dehydrogenase; ATGL*,* Adipose triglyceride lipase; HSL, hormone-sensitive lipase; MAGL*,* monoacylglycerol lipase

**Futile Cycle**	**Tissue**	**Physiological Role**	**Protein Involved**	**ATP-Dependent**	**References**
UCP1-mediated uncoupling	BAT	Thermogenesis	UCP1	**__**	[13, 14, 16, 17, 95]
Calcium cycling	BAT, Skeletal muscle	Thermogenesis	SERCA1, RyR1, SLN	✓	[58–63, 96, 97]
Endogenous mitochondrial uncoupling	BAT	ATP production and thermogenesis	AAC	✓	[45, 46]
Calcium cycling	Beige Fat	Thermogenesis and glucose homeostasis	SERCA2b, RyR2	✓	[24, 34, 52, 53]
Creatine-dependent ADP/ATP cycling	Beige Fat	Thermogenesis	CK, AAC	✓	[21, 64–68]
Glycerolipid-free fatty acid cycle	WAT, BAT, Islet β-cell	Lipolysis and triglyceride synthesis	ATGL, HSL, MAGL, GK, MPC	✓	[70, 71, 74, 79, 83, 84]
Glyceroneogenesis-lipid cycle	Liver, WAT, BAT	G3P formation and triglyceride synthesis	PEPCK-C, Glycerol Kinase	✓	[69, 70, 72, 73, 78]
Cori Cycle	Skeletal Muscle and Liver	Lactate and glucose production	LDH	✓	[92]

Numerous studies have characterized the role and regulation of UCP1-mediated thermogenesis in brown and beige adipose tissue and we will do it a disservice by trying to review it in one paragraph. The role of UCP1 in thermogenesis has been recently reviewed [22, 25]. BAT thermogenesis and UCP1 activity can decrease body fat accumulation, improving insulin sensitivity and glycemic control in diet-induced obese mice [26]. Accordingly, short-term cold exposure (4 h) decreases circulating triglyceride concentrations and increases insulin sensitivity in mice [27]. Moreover, mice with genetic ablation of BAT are more susceptible to diet-induced obesity and insulin resistance [28, 29]. Similarly, mice lacking beige fat function caused by the adipocyte-specific deletion of PRDM16 develop obesity and insulin resistance [30].

It was previously hypothesized that the action of UCP1 primarily mediates the “anti-obesity” and “anti-diabetic” actions of brown and beige fat. Surprisingly, UCP1-knock out mice maintained normal resting energy expenditure and were resistant to diet-induced obesity at sub-thermoneutral temperatures [31–33]. Additionally, it was shown that UCP1-deficient mice can adapt to a cold environment, highlighting the presence of UCP1-independent mechanisms of adaptive thermogenesis and regulation of whole-body energy homeostasis [32, 34]. UCP1-knock out mice are protected from diet-induced obesity at sub-thermoneutral temperatures, because the alternative thermogenic mechanisms consume more calories to produce the same amount of heat needed for thermoregulation [31–33]. Furthermore, deletion of the mitochondrial protein, Mfn2, in brown adipose tissue protected from insulin resistance and obesity, despite impairing cold-induced thermogenesis. In this regard, BAT-specific Mfn2 deletion decreased the animal energy efficiency and increased coupled fat oxidation in BAT, explaining protection from obesity and hyperglycemia [35]. Altogether, these studies suggest that outside UCP1 mediated uncoupling, other mechanisms may affect energy expenditure by changing energy efficiency.

While some of the animal studies were translated to humans, others were not. Earlier clinical observations in humans demonstrated that both cold exposure [19, 36, 37] and treatment with mitochondrial uncouplers [38–40] result in increased energy expenditure and weight loss [41]. More recent BAT-targeted pharmacological studies have failed to demonstrate that BAT activation results in a significant weight loss by increasing overall energy expenditure [42–44]. For example, treatment of human subjects with a B-3 adrenergic receptor agonist Mirabegron did not result in a significant weight loss although it successfully activated thermogenesis by BAT [44]. The lack of success of Mirabegron was hypothesized to be explained by the lower mass of BAT in humans when compared to mice. An alternative hypothesis could be that increased energy expenditure by adaptive thermogenesis is compensated for by an increase in energy efficiency in other tissues, leading to an overall unchanged or reduced BMR.

## UCP1-independent mechanisms of mitochondrial uncoupling

**Fig. 1:**
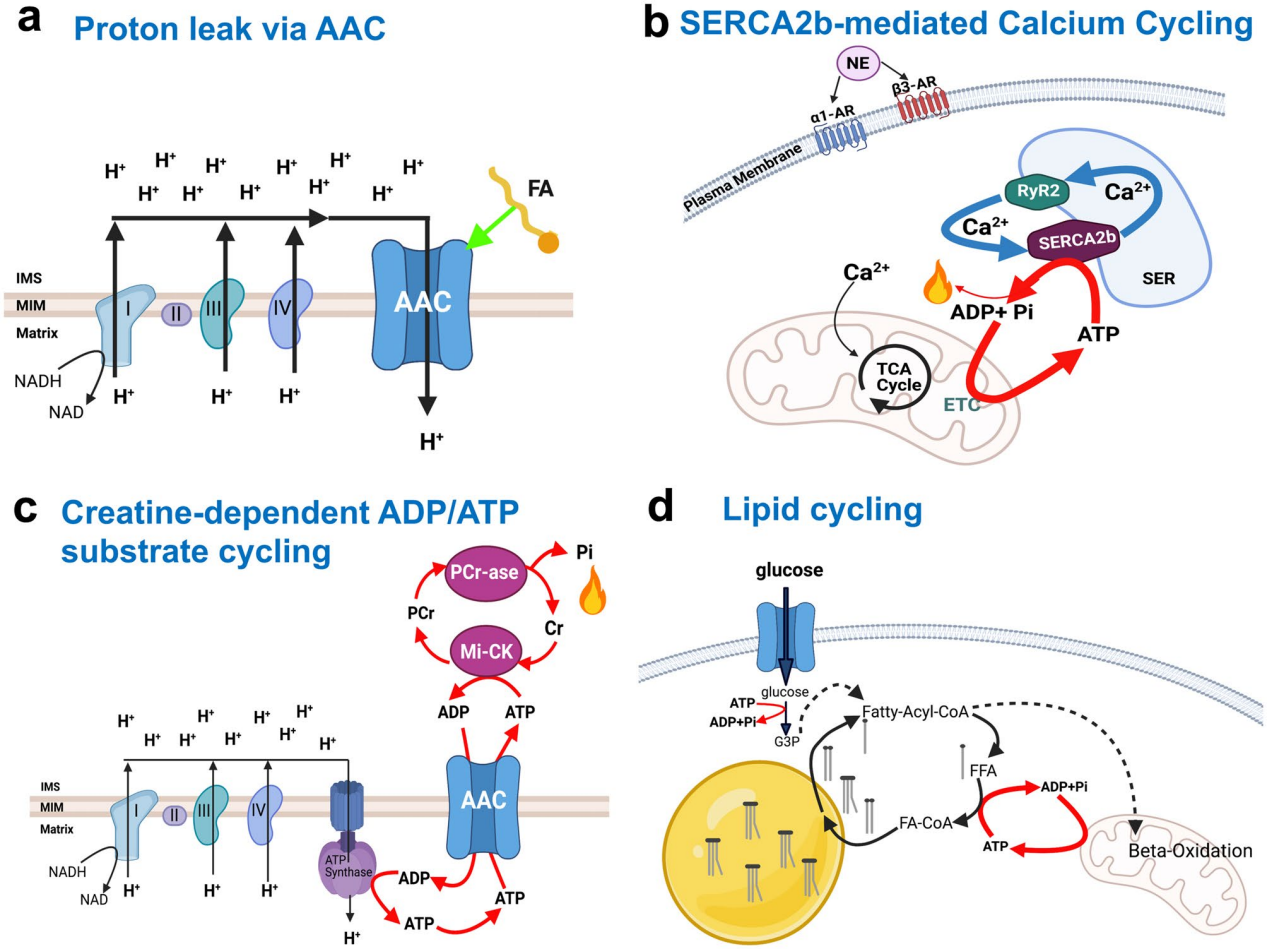
ATP-Dependent Futile Cycles. (**a**) The mitochondrial ADP/ATP carrier (AAC) can act as H + transporter, in addition to its function as ADP/ATP exchanger. AAC-mediated proton leak requires the presence of free fatty acids and is negatively regulated by ADP/ATP exchange. Thereby, AAC provides an alternative mechanism to induce mitochondrial proton leak. (**b**) In beige adipocytes norepinephrine (NE)–mediated stimulation of adrenergic receptors stimulates futile Ca2 + cycling through activation of sarco/endoplasmic reticulum Ca^2+^-ATPase 2b (SERCA2b) and ryanodine receptor 2 (RyR2). SER, sarco/endoplasmic reticulum. (**c**) In UCP1-deficient adipocytes creatine (Cr) and creatine and CK-mediated hydrolysis of ATP stimulates cycling of ATP production and consumption when ADP is limiting through the ATP/ADP carrier (AAC). The mitochondrial AAC localizes to the mitochondrial inner membrane and functions as an ADP/ATP exchanger to control the cellular ATP pool. MI-CK, mitochondrial-creatine kinase; PCr, phosphor-creatine; PCr-ase, phosphor-creatine kinase. (**d**) In the absence of mitochondrial pyruvate import, brown adipocytes activate lipolysis, which induces futile lipid cycling and β-oxidation. Additional details about the malate aspartate shuttle as a mechanism to induce lipid cycling appear in the text. FFA, free fatty acid; G3P, glycerol-3-phosphate

The mitochondrial ADP/ATP carrier, (AAC), acts as a mitochondrial uncoupler under certain conditions, fueling a futile cycle of proton diffusion across the inner mitochondrial membrane [45] (Fig. 1, Table 1). AAC is a member of the solute carrier family (SLC25) that exchanges ATP and ADP between the mitochondria matrix and the cytosol [45]. However, AAC has additional key functions: AAC is a fatty-acid induced proton channel that explains endogenous proton leak, as well as regulating the opening of the permeability transition pore and the removal of mitochondria by mitophagy [45, 46]. Remarkably, both AAC-controlled leak and PTP opening can be stimulated by free fatty acids, similarly to how fatty acids activate UCP1 [45]. This connection by fatty acids highlights the importance of nutrients as active regulators of energy dissipation, by determining the relative proportion that is directed to mitochondrial ATP synthesis versus leak.

Another mechanism that uncouples oxygen consumption from ATP synthesis by allowing protons to enter the mitochondrial matrix is mediated by the mitochondrial Nicotinamide nucleotide transhydrogenase (NNT) [47–49]. NNT uses the electrons in NADH to reduce NADP^+^ to NADPH, with this transhydrogenation being coupled to proton translocation across the inner membrane [47–49]. The energy consumed by NNT-mediated proton translocation generates NADPH. Therefore, although the NNT uncouples the mitochondria respiration from ATP synthesis, energy consumed by the NNT is not directly transformed into heat and thus will not be dissipated out of the organism [50].

## The two categories of thermogenic energy expenditure: ATP-consuming futile cycles and uncoupling

Recent evidence demonstrated that BAT and beige adipocytes can increase energy expenditure by activating ATP-consuming futile cycles. This need for ATP means that mitochondria can remain coupled to sustain ATP demand and fuel these futile cycles, which prevents the potential risks associated with large depolarization characteristic of mitochondrial uncoupling (Table 1). Remarkably, these ATP-consuming futile cycles recently identified in BAT and beige adipocytes were already shown to promote energy expenditure in skeletal muscle.

A futile cycle consists of a set of biochemical reactions that concurrently run in opposite directions, consuming ATP in one of the directions. As the product of the first reaction is the substrate of the second reaction, and the product of the second reaction is the substrate of the first reaction, such a cycle will lead to a net decrease in ATP [51]. While we use the word futile, this does not mean there is no use for these cycles. Outcomes of futile cycles include the consumption of excess nutrients and the generation of heat. Furthermore, futile cycles are characterized by the continuous production of the cycle’s intermediates allowing for the maintenance of a steady pool of metabolites comprising the cycle and the active enzymes producing them. As such, futile cycles allow for a quick response to cellular events requiring the production or processing of the cycle intermediates.

The capacity of energy-wasting mechanisms based on futile cycles to decrease body weight loss will be mostly dependent on their abundancy and/or frequency of execution. Therefore, the extent of energy expenditure achieved by each of these mechanisms per cell at a given time is unlikely to be a reliable predictor of their impact on weight loss.

## Calcium futile cycles in beige and brown adipocytes

A UCP1-independent thermogenic pathway that involves an ATP-consuming Ca^2+^ futile cycling was shown to contribute to beige fat energy expenditure and systemic glucose homeostasis (Fig. 1, Table 1) [24, 34, 52]. In response to cold exposure, NE binds to α1-AR (adrenergic receptor) and β3-AR to increase intracellular Ca^2+^ cycling by activating the ryanodine receptor 2 (RyR2) and promoting the extrusion of ER-calcium. Calcium extrusion then increases the activity of the sarco(endo) plasmic reticulum Ca^2+^ (SERCA2b), which brings the calcium back to the ER of beige adipocytes [24, 52, 53]. As SERCA2b consumes ATP to transport calcium back to the ER, the activation of this calcium cycle leads to a futile consumption of ATP that is responsible for energy dissipation. In addition, decreased efficiency of SERCA2b importing calcium leads to an increase in Ca^2+^ levels in the cytosol, which enter the mitochondria to activate pyruvate dehydrogenase activity and ATP synthesis [24, 53]. Thus, calcium itself activates mitochondria to cover this increase ATP demand more efficiently. This calcium cycle explained why UCP1 KO beige adipocytes utilize glucose-derived pyruvate as the primary fuel source for mitochondrial respiration [24, 53]. In all, SERCA2-mediated calcium cycling increases energy dissipation while elevating glucose oxidation, which counteracts hyperglycemia [24].

Intriguingly, the SERCA2b-mediated Ca^2+^ cycling thermogenic mechanism is necessary for beige adipocyte thermogenesis but is dispensable in brown adipocytes that express high levels of UCP1. It is hypothesized that this pathway is unique to beige fat due to the high expression of ATP synthase not found in brown adipose tissue, enabling beige fat to dissipate energy by increasing ATP consumption in a futile manner [54–56].

Although regulators of SERCA2 activity in beige adipocytes have yet to be established, the regulatory protein sarcolipin (SLN) has been shown to promote uncoupling of ATP hydrolysis of SERCA1a from calcium transport in skeletal muscle, resulting in muscle nonshivering thermogenesis [57–59]. Unphosphorylated SLN induces conformational changes in SERCA structure which decreases the affinity of SERCA to bind calcium [57]. This decrease in affinity causes SERCA to hydrolyze more ATP to transport less calcium, meaning that more ATP will be consumed to perform the same transport process. Accordingly, it was shown that SLN enhances energy dissipation and heat production in skeletal muscle, concurrent with a sustained elevation of cytoplasmic Ca^2+^.

Interestingly, SERCA1 was recently identified in the inner mitochondrial membranes (IMM) of BAT and was shown to induce SERCA/RyR mediated Ca^2+^ futile cycling in BAT mitochondria (Table 1) [60]. Calcium is pumped from the matrix to the inner mitochondrial space (IMS) by SERCA1 and returns to the mitochondrial matrix through the RyR [61–63]. The ATP hydrolysis activity of SERCA1 was shown to absorb part of the ATP derived from the electron flux activated by Ca^2+^, contributing to brown adipose tissue energy expenditure [63].

## Creatine-phosphocreatine futile cycle

A Creatine-dependent ATP-consuming cycle has been shown to promote energy dissipation in beige fat and brown adipocytes (Fig. 1, Table 1) [21, 64–68]. This cycle was initially identified in mitochondria isolated from beige fat of cold-exposed animals under ADP-limited conditions and was further confirmed to exist in both mouse and human brown adipocytes [21, 65]. Preliminary studies demonstrated that creatine stimulates substrate cycling and increases ADP-dependent respiration in beige fat mitochondria when ADP is limiting [21]. When UCP1 is deleted, genes involved in creatine metabolism including creatine kinase B (CKB) are upregulated by thermogenic cAMP signaling [21, 65]. Moreover, depleting creatine levels in thermogenic adipocytes of mice by deleting the rate-limiting enzyme of creatine biosynthesis, glycine amidinotransferase (GATM), impairs energy expenditure due to reduced thermogenesis and causes diet-induced obesity [67, 68]. The existence of this cycle in BAT supports the concept that increasing mitochondrial ATP synthesis can be used as an approach to promote energy dissipation.

## Lipid cycling as a futile cycle consuming ATP

Lipid cycling consists of a catabolic segment and an anabolic segment. The catabolic segment refers to the hydrolysis of triglycerides (TAGs) into free fatty acids (FFAs) and glycerol [69–71]. Adipose triglyceride lipase (ATGL), hormone-sensitive lipase (HSL), and monoacylglycerol lipase (MAGL) are the main enzymes catalyzing this hydrolysis, namely lipolysis. The free glycerol that is generated during the breakdown of triglycerides can potentially be reused by the anabolic segment, constituted by glycerol kinase, which consumes ATP to regenerate glycerol-3-phosphate that can be utilized to rebuild triglycerides [70]. In cells with low glycerol kinase activity, glycerol can be released into the bloodstream, preventing this ATP-consuming lipid cycle [69]. However, glucose can be consumed to make new triglycerides by fueling glyceroneogenesis, a process controlled by phosphoenolpyruvate carboxykinase (GTP) (PEPCK-C) (Table 1) [69, 70, 72]. Glyceroneogenesis is active in liver, white and brown adipose tissue. Therefore, re-esterification and futile ATP consumption can still occur in cells with low glycerol kinase activity via PEPCK-C. In BAT, glyceroneogenesis has an important role in determining the rate of triglyceride re-esterification after norepinephrine stimulation [73]. It has been reported that cold exposure promotes a decrease in PEPCK-C in BAT, which is accompanied by a decrease in re-esterification as the FFAs need to be delivered to the mitochondria to maintain thermogenesis [73].

Glycerol metabolism is not the only ATP-consuming process that can increase energy dissipation in adipocytes. Glycerolipid-free fatty acid cycling (GL/FFA or lipid cycling) is a futile cycle that consumes ATP via the partial or full re-esterification of free fatty acids into TAGs, diacylglycerols (DAGs), or Monoacylglycerols (MAGs) (Table 1) [70, 71]. The reason is that lipolysis removes CoA from fatty acids and the addition of CoA to fatty acids consumes ATP, with CoA addition to FFA being essential for esterification. Each GL/FFA cycle consumes 7 ATP molecules [74].

**Fig. 2:**
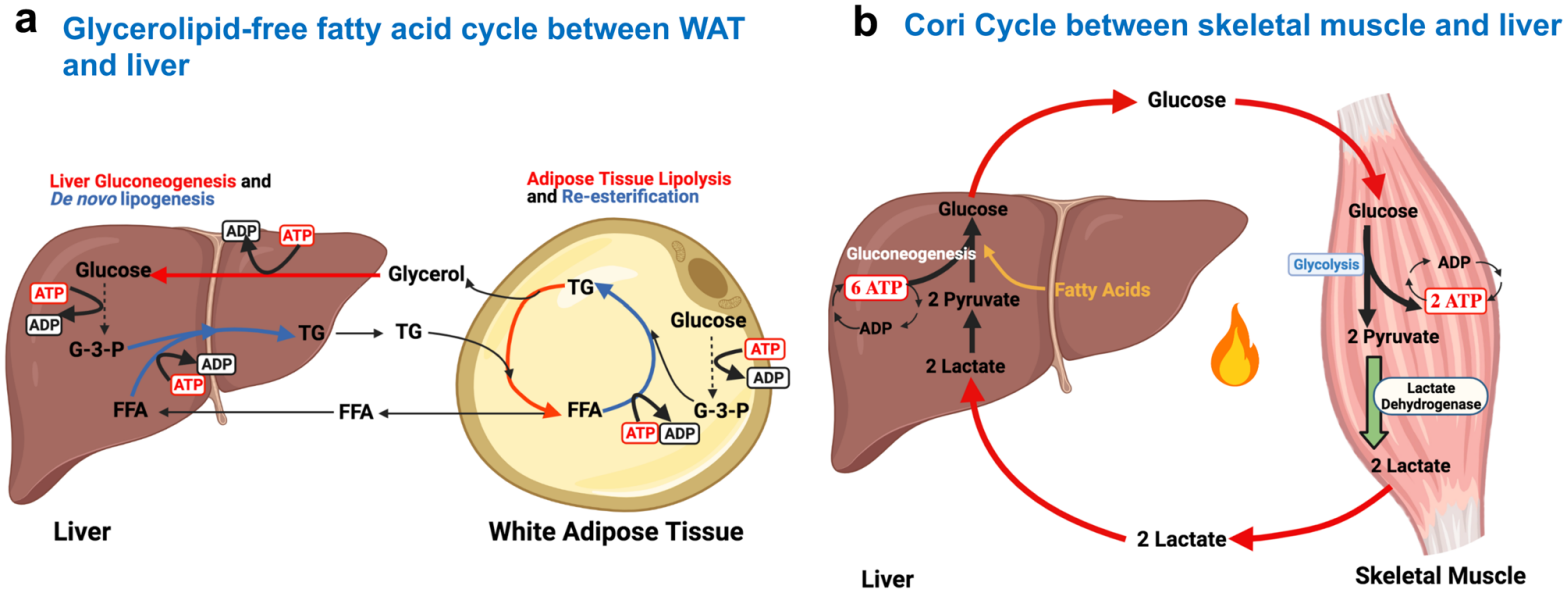
Futile Cycles That Connect Multiple Subcellular Compartments and Tissues. (**a**) Glycerolipid-free fatty acid cycle between white adipose tissue and the liver. Triglycerides (TGs) are broken down to glycerol and free fatty acids (FFAs), which are either re-esterified in the adipose tissue or released into the bloodstream. The liver converts the glycerol from the bloodstream to glucose through gluconeogenesis, and this glucose can then be used to make glycerol-3-phosphate (G-3-P) needed for triglyceride synthesis. Free fatty acids picked up by the liver are used along with glycerol-3-phosphate for *de novo* triglyceride synthesis also called *de novo* lipogenesis. (**b**) Cori cycle between skeletal muscle and the liver. Glucose in the muscle is metabolized to pyruvate and ATP through glycolysis and then converted to lactate by lactate dehydrogenase (LDH), which is released into the blood and picked up by the liver. The liver uses the lactate to produce glucose, utilizing ATP, and the glucose is then released back into the circulation. Overall this futile cycle results in a net loss of 4 ATPs

The functional role of lipid cycling can range from facilitating a rapid provision of FFA for oxidation, to cell signaling regulation and detoxification from excess free fatty acids [75]. Importantly, the anabolic and catabolic segments of lipid cycling can concurrently occur in the same cell or in different tissues; for example between the liver and white adipose tissue (Fig. 2). Abnormalities in the function of lipid cycling are associated with insulin resistance, type 2 diabetes, fatty liver disease, and cancer [71, 76, 77].

A significant amount of basal energy expenditure in white adipose tissue is attributed to lipid cycling [78]. The reason is that mature white adipocytes do not oxidize fatty acids but are a major source of free fatty acids for surrounding tissues [79–81]. In cultured white adipocytes from humans, around 40% of released fatty acids are recycled back into lipid droplets [79]. *In vivo*, Wolfe et al. showed that in humans at rest around 70% of fatty acids are re-esterified in the adipose tissue [82]. Especially at the beginning of physical exercise, re-esterification is reduced to 25%, suggesting that decreased re-esterification is explained by fatty acids consumption in muscle and liver [82]. This suggests that lipid cycling enables the adipose tissue to be ready to deliver oxidizable fatty acids to muscle and liver, while the esterification component protects the adipocytes from lipotoxicity when fatty acids are not oxidized [82].

## Mechanisms inducing lipid cycling

While the majority of heat in activated brown adipocytes is generated via UCP1 mediated proton leak, part of thermogenesis is attributed to lipid cycling [83]. Recently, Veliova et al. proposed a novel mechanism to activate lipid cycling and increase energy dissipation in non-stimulated brown adipocytes, by blocking the mitochondrial pyruvate carrier (MPC) (Fig. 1) [84]. Inhibition of the MPC results in increased ATP demand and coupled mitochondrial fat oxidation to support fatty acid esterification, as energy expenditure was sensitive to the ACS inhibitor Triacsin C. Consequently, MPC blockage increases fat oxidation and energy expenditure bypassing the need for adrenergic stimulation or mitochondrial uncoupling to oxidize fat [84]. It remains to be determined whether lipid cycling can be induced in white adipocytes by a similar mechanism, which would be particularly interesting as a way to promote energy-wasting and possibly weight loss.

Aside from its role in maintaining the availability of free fatty acids, lipid cycling has an essential role in cell signaling, in pancreatic beta cells, lipid species can act as signaling molecules that modulate insulin secretion [70, 74, 85]. Furthermore, the increased energy demand caused by lipid cycling has been linked to AMPK activation in adipocytes and beta cells, as lipid cycling can increase the AMP/ATP ratio [74, 86]. In addition, lipid cycling can play an important role in signaling from fatty acids and glucose to promote cell growth and modulate gene expression [87]. It was proposed that high glycolysis rates that support cancer proliferation could be maintained by Glycerol-3-phosphate dehydrogenase (GPDH), which regenerates NAD + by transforming G-3-P to DHAP [88].

Moreover, it has been demonstrated that the type 2 diabetes drugs, Thiazolidinediones (TZDs), which are agonists for peroxisome proliferator-activated receptor-gamma (PPARγ) and block MPC activity, induce changes in fatty acid esterification [89]. TZDs induced the expression of PEPCK, a key regulator of the glyceroneogenesis pathway [89]. The activation of glyceroneogenesis in adipose tissue by TZDs allows the re-esterification of fatty acids via lipid cycling, thus lowering fatty acid release into the plasma [89]. Importantly, reduced fatty acid levels in the plasma may be important for the insulin-sensitizing action of TZDs [89]. However, whether some of the actions on fatty acid cycling induced by TZDs are mediated by their actions on MPC has not been characterized.

## Futile cycles that connect multiple subcellular compartments and tissues

To avoid uncontrolled dissipation of energy, some futile cycles have their core steps occurring in different tissues or even in different subcellular compartments. An example is the hexokinase/glucose‐6‐phosphatase reaction, wherein the liver hexokinase is replaced by glucokinase (GK), which is regulated by glucokinase regulatory protein (GKRP) [90, 91]. Under fasting, GKRP inactivates and sequesters GK in the nucleus to prevent futile cycles of glucose phosphorylation during gluconeogenesis [90, 91].

Interestingly, the same mechanism used to induce lipid cycling in adipose tissue was shown to contribute to the Cori cycle that communicates between muscle and liver (Fig. 2). Skeletal muscle-specific deletion of the mitochondrial pyruvate carrier (MPC) in mice increases flux through the Cori cycle, leading to an increase in whole-body energy expenditure [92]. Muscle-specific MPC deletion (MPC SkmKO) promoted the conversion of pyruvate to lactate in the muscle and its release of lactate into the circulation [92]. This lactate provides carbons for glucose production in the liver, which resupplies glucose to the muscle. The Cori cycle is futile because each round produces two skeletal muscle ATP molecules and consumes six in the liver, for a net consumption of 4 ATP equivalents. Cori cycling is energetically supported by fatty acid oxidation in the liver for gluconeogenesis, as well as in the muscle to support muscle ATP demand. This increase in mitochondrial fat oxidation was shown to account for a decrease in body weight in mice with skeletal muscle-specific deletion of the MPC [92].

## Conclusions

Energy homeostasis is maintained by a balance of energy intake and energy dissipation. The basal metabolic rate can be regulated to decrease energy dissipation during periods of decreased nutrient intake. The existence of these compensatory mechanisms means that cells can fine-tune the amount of energy required to sustain the same essential metabolic processes. Understanding the regulation of energy-dissipating processes modulated by food intake can potentially lead to therapeutic strategies that serve to promote weight loss among individuals with obesity.

It has been shown through the use of chemical uncouplers that increasing the metabolic rate can cause weight loss and supports the hypothesis that increasing energy expenditure by decreasing metabolic efficiency is a potential mechanism to induce a negative energy balance [39, 40]. However, chemical uncouplers and adrenergic inducers of thermogenesis have several disadvantages and risks related to their safety, including their lack of tissue specificity and the potential disruption of other functions of mitochondria that are membrane potential-dependent [93, 94]. Specifically, treatment with uncouplers can result in large enough mitochondrial depolarization that will impair charge-dependent transport processes required for metabolite import as well as calcium buffering. Depolarization can also increase the propensity for permeability transition and apoptosis. On the other hand, mechanisms that increase ATP demand through futile cycles dissipate energy through pathways that can be more easily made tissue-specific and that are less disruptive to mitochondrial functions. By increasing ATP demand, ATP-consuming futile cycles can induce mild depolarization that is unlikely to affect other mitochondrial functions. Conversely, whether the adaptive response of the organisms to the induction of futile cycles may consist of either transient or persistent change in BMR and sustainable reduction in weight remains to be seen.

In conditions where ATP production is compromised, an ATP-consuming futile cycle can be deleterious. By blocking ATP-consuming futile cycles, it is possible we can prevent cell death or dysfunctionality in conditions such as ischemia and mitochondrial respiratory chain diseases, where ATP demand exceeds the synthesis rate.

Overall, ATP-consuming futile cycles hold promise both as an intervention to treat obesity-related diseases as well as conditions of unmet ATP demand. Further elucidating mechanisms that control ATP-consuming futile cycles may allow for the development of drugs that can modify the ATP demand in a tissue-specific manner.

## Abbreviations

WAT: White adipose tissue
BAT: Brown adipose tissue
FFAs: Free fatty acids
NE: Norepinephrine
UCP1: Uncoupling protein 1
BMI: Body mass index
^18^F-FDG: ^18^F-fluorodeoxyglucose
PET: Positron emission tomography
CT: Computed tomography
ETC: Electron transport chain
AAC: Mitochondrial ADP/ATP carrier
SERCA1/2b: Sarco/endoplasmic reticulum Ca^2^^+^-ATPase
PEPCK-C: Phosphoenolpyruvate carboxykinase
GL/FFA or lipid cycling: Glycerolipid—free fatty acid cycling
G3P: Glycerol-3-phosphate
TGs: Triglycerides
DAGs: Diacylglycerols
MAGs: Monoacylglycerols
ATGL: Adipose triglyceride lipase
HSL: Hormone-sensitive lipase
MAGL: Monoacylglycerol lipase
DHAP: Dihydroxyacetone phosphate
GPDH: Glycerol-3-phosphate dehydrogenase
RyR2: Ryanodine receptor 2
β_2_-AR: β_2_-Adrenergic receptor
β_3_-AR: β_3_-Adrenergic receptor
SLN: Sarcolipin
CK: Creatine kinase
PCr: Phosphocreatine
MaSH: Malate-aspartate shuttle
LPA: Lysophosphatidic
αKG: α-Ketoglutarate
OA: Oxaloacetate
Glu: Glutamate
Asp: Aspartate
MPC: Mitochondrial pyruvate carrier
LDH: Lactate dehydrogenase
NNT: Nicotinamide nucleotide transhydrogenase
TZDs: Thiazolidinediones

## References

[1] Leibel RL, Rosenbaum M, Hirsch J (1995). Changes in energy expenditure resulting from altered body weight. N Engl J Med.

[2] Fothergill E, Guo J, Howard L, Kerns JC, Knuth ND, Brychta R (2016). Persistent metabolic adaptation 6 years after The Biggest Loser competition. Obesity (Silver Spring).

[3] Johannsen DL, Knuth ND, Huizenga R, Rood JC, Ravussin E, Hall KD (2012). Metabolic slowing with massive weight loss despite preservation of fat-free mass. J Clin Endocrinol Metab.

[4] Dulloo AG, Calokatisa R (1991). Adaptation to low calorie intake in obese mice: contribution of a metabolic component to diminished energy expenditures during and after weight loss. Int J Obes.

[5] Valtueña S, Blanch S, Barenys M, Solà R, Salas-Salvadó J (1995). Changes in body composition and resting energy expenditure after rapid weight loss: is there an energy-metabolism adaptation in obese patients?. Int J Obes Relat Metab Disord.

[6] Elliot DL, Goldberg L, Kuehl KS, Bennett WM (1989). Sustained depression of the resting metabolic rate after massive weight loss. Am J Clin Nutr.

[7] Kaiyala KJ, Morton GJ, Leroux BG, Ogimoto K, Wisse B, Schwartz MW (2010). Identification of body fat mass as a major determinant of metabolic rate in mice. Diabetes.

[8] Corbett SW, Stern JS, Keesey RE (1986). Energy expenditure in rats with diet-induced obesity. Am J Clin Nutr.

[9] Maclean PS, Bergouignan A, Cornier MA, Jackman MR (2011). Biology's response to dieting: the impetus for weight regain. Am J Physiol Regul Integr Comp Physiol.

[10] Dulloo AG, Jacquet J (1998). Adaptive reduction in basal metabolic rate in response to food deprivation in humans: a role for feedback signals from fat stores. Am J Clin Nutr.

[11] Himms-Hagen J (1984). Nonshivering thermogenesis. Brain Res Bull.

[12] Cypess AM, Lehman S, Williams G, Tal I, Rodman D, Goldfine AB (2009). Identification and importance of brown adipose tissue in adult humans. N Engl J Med.

[13] Fedorenko A, Lishko PV, Kirichok Y (2012). Mechanism of fatty-acid-dependent UCP1 uncoupling in brown fat mitochondria. Cell.

[14] Parker N, Crichton PG, Vidal-Puig AJ, Brand MD (2009). Uncoupling protein-1 (UCP1) contributes to the basal proton conductance of brown adipose tissue mitochondria. J Bioenerg Biomembr.

[15] Jastroch M, Divakaruni AS, Mookerjee S, Treberg JR, Brand MD (2010). Mitochondrial proton and electron leaks. Essays Biochem.

[16] Foster DO, Frydman ML (1978). Brown adipose tissue: the dominant site of nonshivering thermogenesis in the rat. Experientia Suppl.

[17] Rothwell NJ, Stock MJ (1979). A role for brown adipose tissue in diet-induced thermogenesis. Nature.

[18] Cannon B, Nedergaard J (2004). Brown adipose tissue: function and physiological significance. Physiol Rev.

[19] Saito M, Okamatsu-Ogura Y, Matsushita M, Watanabe K, Yoneshiro T, Nio-Kobayashi J (2009). High incidence of metabolically active brown adipose tissue in healthy adult humans: effects of cold exposure and adiposity. Diabetes.

[20] Cohen P, Spiegelman BM (2015). Brown and beige fat: molecular parts of a thermogenic machine. Diabetes.

[21] Kazak L, Chouchani ET, Jedrychowski MP, Erickson BK, Shinoda K, Cohen P (2015). A creatine-driven substrate cycle enhances energy expenditure and thermogenesis in beige fat. Cell.

[22] Chouchani ET, Kazak L, Spiegelman BM (2019). New Advances in Adaptive Thermogenesis: UCP1 and Beyond. Cell Metab.

[23] Ikeda K, Yamada T (2020). UCP1 dependent and independent thermogenesis in brown and beige adipocytes. Front Endocrinol (Lausanne).

[24] Ikeda K, Kang Q, Yoneshiro T, Camporez JP, Maki H, Homma M (2017). UCP1-independent signaling involving SERCA2b-mediated calcium cycling regulates beige fat thermogenesis and systemic glucose homeostasis. Nat Med.

[25] Nedergaard J, Cannon B (2018). Brown adipose tissue as a heat-producing thermoeffector. Handb Clin Neurol.

[26] Stanford KI, Middelbeek RJ, Townsend KL, An D, Nygaard EB, Hitchcox KM (2013). Brown adipose tissue regulates glucose homeostasis and insulin sensitivity. J Clin Invest.

[27] Bartelt A, Bruns OT, Reimer R, Hohenberg H, Ittrich H, Peldschus K (2011). Brown adipose tissue activity controls triglyceride clearance. Nat Med.

[28] Hamann A, Benecke H, Le Marchand-Brustel Y, Susulic VS, Lowell BB, Flier JS (1995). Characterization of insulin resistance and NIDDM in transgenic mice with reduced brown fat. Diabetes.

[29] Lowell BB, Flier JS (1997). Brown adipose tissue, β3-Adrenergic receptors, and obesity. Annu Rev Med.

[30] Cohen P, Levy JD, Zhang Y, Frontini A, Kolodin DP, Svensson KJ (2014). Ablation of PRDM16 and beige adipose causes metabolic dysfunction and a subcutaneous to visceral fat switch. Cell.

[31] Enerbäck S, Jacobsson A, Simpson EM, Guerra C, Yamashita H, Harper ME (1997). Mice lacking mitochondrial uncoupling protein are cold-sensitive but not obese. Nature.

[32] Liu X, Rossmeisl M, McClaine J, Riachi M, Harper ME, Kozak LP (2003). Paradoxical resistance to diet-induced obesity in UCP1-deficient mice. J Clin Invest.

[33] Stefl B, Janovská A, Hodný Z, Rossmeisl M, Horáková M, Syrový I (1998). Brown fat is essential for cold-induced thermogenesis but not for obesity resistance in aP2-Ucp mice. Am J Physiol.

[34] Ukropec J, Anunciado RP, Ravussin Y, Hulver MW, Kozak LP (2006). UCP1-independent thermogenesis in white adipose tissue of cold-acclimated Ucp1-/- mice. J Biol Chem.

[35] Mahdaviani K, Benador IY, Su S, Gharakhanian RA, Stiles L, Trudeau KM (2017). Mfn2 deletion in brown adipose tissue protects from insulin resistance and impairs thermogenesis. EMBO Rep.

[36] Yoneshiro T, Aita S, Matsushita M, Kameya T, Nakada K, Kawai Y (2011). Brown adipose tissue, whole-body energy expenditure, and thermogenesis in healthy adult men. Obesity (Silver Spring).

[37] Ouellet V, Labbé SM, Blondin DP, Phoenix S, Guérin B, Haman F (2012). Brown adipose tissue oxidative metabolism contributes to energy expenditure during acute cold exposure in humans. J Clin Invest.

[38] Cutting WC, Mehrtens HG, Tainter ML. Actions and Uses of Dinitrophenol: Promising Metabolic Applications. JAMA. 1933;101(3):193–195. 10.1001/jama.1933.02740280013006.

[39] Tainter ML, Cutting WC, Stockton AB (1934). Use of dinitrophenol in nutritional disorders: a critical survey of clinical results. Am J Public Health Nations Health.

[40] Parascandola J (1974). Dinitrophenol and bioenergetics: an historical perspective. Mol Cell Biochem.

[41] Yoneshiro T, Saito M (2015). Activation and recruitment of brown adipose tissue as anti-obesity regimens in humans. Ann Med.

[42] Larson CJ (2019). Translational pharmacology and physiology of brown adipose tissue in human disease and treatment. Handb Exp Pharmacol.

[43] Chen KY, Brychta RJ, Abdul Sater Z, Cassimatis TM, Cero C, Fletcher LA (2020). Opportunities and challenges in the therapeutic activation of human energy expenditure and thermogenesis to manage obesity. J Biol Chem.

[44] O'Mara AE, Johnson JW, Linderman JD, Brychta RJ, McGehee S, Fletcher LA (2020). Chronic mirabegron treatment increases human brown fat, HDL cholesterol, and insulin sensitivity. J Clin Invest.

[45] Bertholet AM, Chouchani ET, Kazak L, Angelin A, Fedorenko A, Long JZ (2019). H(+) transport is an integral function of the mitochondrial ADP/ATP carrier. Nature.

[46] Kreiter J, Rupprecht A, Škulj S, Brkljača Z, Žuna K, Knyazev DG, et al. ANT1 activation and inhibition patterns support the fatty acid cycling mechanism for proton transport. Int J Mol Sci. 2021;22(5). 10.3390/ijms22052490. 10.3390/ijms22052490 PMC7958136 33801254

[47] Jackson JB, Leung JH, Stout CD, Schurig-Briccio LA, Gennis RB. Review and hypothesis. New insights into the reaction mechanism of transhydrogenase: swivelling the dIII component may gate the proton channel. FEBS Lett. 2015;589(16):2027–33. 10.1016/j.febslet.2015.06.027. 10.1016/j.febslet.2015.06.027 26143375

[48] Kampjut D, Sazanov LA (2019). Structure and mechanism of mitochondrial proton-translocating transhydrogenase. Nature.

[49] Hoek JB, Rydström J (1988). Physiological roles of nicotinamide nucleotide transhydrogenase. Biochem J.

[50] Rydström J (2006). Mitochondrial transhydrogenase–a key enzyme in insulin secretion and potentially, diabetes. Trends Biochem Sci.

[51] Newsholme EA (1978). Substrate cycles: their metabolic, energetic and thermic consequences in man. Biochem Soc Symp.

[52] Gamu D, Tupling AR (2017). Fattening the role of Ca(2+) cycling in adaptive thermogenesis. Nat Med.

[53] Mottillo EP, Ramseyer VD, Granneman JG (2018). SERCA2b cycles its way to UCP1-Independent thermogenesis in beige fat. Cell Metab.

[54] Cannon B, Vogel G. The mitochondrial ATPase of brown adipose tissue. Purification and comparison with the mitochondrial ATPase from beef heart. FEBS Lett. 1977;76(2):284–9. 10.1016/0014-5793(77)80169-5. 10.1016/0014-5793(77)80169-5 140818

[55] Lindberg O, de Pierre J, Rylander E, Afzelius BA (1967). Studies of the mitochondrial energy-transfer system of brown adipose tissue. J Cell Biol.

[56] Ikeda K, Maretich P, Kajimura S (2018). The common and distinct features of brown and beige adipocytes. Trends Endocrinol Metab.

[57] Autry JM, Thomas DD, Espinoza-Fonseca LM (2016). Sarcolipin promotes uncoupling of the SERCA Ca(2+) pump by inducing a structural rearrangement in the energy-transduction domain. Biochemistry.

[58] Bal NC, Maurya SK, Sopariwala DH, Sahoo SK, Gupta SC, Shaikh SA (2012). Sarcolipin is a newly identified regulator of muscle-based thermogenesis in mammals. Nat Med.

[59] Sahoo SK, Shaikh SA, Sopariwala DH, Bal NC, Periasamy M (2013). Sarcolipin protein interaction with sarco(endo)plasmic reticulum Ca2+ ATPase (SERCA) is distinct from phospholamban protein, and only sarcolipin can promote uncoupling of the SERCA pump. J Biol Chem.

[60] de Meis L (2003). Brown adipose tissue Ca2+-ATPase: uncoupled ATP hydrolysis and thermogenic activity. J Biol Chem.

[61] de Meis L, Oliveira GM, Arruda AP, Santos R, Costa RM, Benchimol M (2005). The thermogenic activity of rat brown adipose tissue and rabbit white muscle Ca2+-ATPase. IUBMB Life.

[62] de Meis L, Arruda AP, da Costa RM, Benchimol M (2006). Identification of a Ca2+-ATPase in brown adipose tissue mitochondria: regulation of thermogenesis by ATP and Ca2+. J Biol Chem.

[63] de Meis L, Ketzer LA, da Costa RM, de Andrade IR, Benchimol M (2010). Fusion of the endoplasmic reticulum and mitochondrial outer membrane in rats brown adipose tissue: activation of thermogenesis by Ca2+. PLoS ONE.

[64] Bertholet AM, Kazak L, Chouchani ET, Bogaczyńska MG, Paranjpe I, Wainwright GL (2017). Mitochondrial patch clamp of beige adipocytes reveals UCP1-Positive and UCP1-Negative cells both exhibiting futile creatine cycling. Cell Metab.

[65] Rahbani JF, Roesler A, Hussain MF, Samborska B, Dykstra CB, Tsai L, et al. Creatine kinase B controls futile creatine cycling in thermogenic fat. 10.1038/s41586-021-03221-y PMC8647628 33597756

[66] Sun Y, Rahbani JF, Jedrychowski MP, Riley CL, Vidoni S, Bogoslavski D (2021). Mitochondrial TNAP controls thermogenesis by hydrolysis of phosphocreatine. Nature.

[67] Kazak L, Chouchani ET, Lu GZ, Jedrychowski MP, Bare CJ, Mina AI (2017). Genetic depletion of adipocyte creatine metabolism inhibits diet-induced thermogenesis and drives obesity. Cell Metab.

[68] Kazak L, Rahbani JF, Samborska B, Lu GZ, Jedrychowski MP, Lajoie M (2019). Ablation of adipocyte creatine transport impairs thermogenesis and causes diet-induced obesity. Nat Metab.

[69] Guan HP, Li Y, Jensen MV, Newgard CB, Steppan CM, Lazar MA (2002). A futile metabolic cycle activated in adipocytes by antidiabetic agents. Nat Med.

[70] Reshef L, Olswang Y, Cassuto H, Blum B, Croniger CM, Kalhan SC (2003). Glyceroneogenesis and the triglyceride/fatty acid cycle. J Biol Chem.

[71] Prentki M, Madiraju SR (2008). Glycerolipid metabolism and signaling in health and disease. Endocr Rev.

[72] Ballard FJ, Hanson RW, Leveille GA (1967). Phosphoenolpyruvate carboxykinase and the synthesis of glyceride-glycerol from pyruvate in adipose tissue. J Biol Chem.

[73] Feldman D, Hirst M (1978). Glucocorticoids and regulation of phosphoenolpyruvate carboxykinase activity in rat brown adipose tissue. Am J Physiol.

[74] Prentki M, Madiraju SR (2012). Glycerolipid/free fatty acid cycle and islet β-cell function in health, obesity and diabetes. Mol Cell Endocrinol.

[75] Chouchani ET, Kajimura S (2019). Metabolic adaptation and maladaptation in adipose tissue. Nat Metab.

[76] Larter CZ, Chitturi S, Heydet D, Farrell GC. A fresh look at NASH pathogenesis. Part 1: the metabolic movers. J Gastroenterol Hepatol. 2010;25(4):672–90. 10.1111/j.1440-1746.2010.06253.x. 10.1111/j.1440-1746.2010.06253.x 20492324

[77] Nomura DK, Long JZ, Niessen S, Hoover HS, Ng SW, Cravatt BF (2010). Monoacylglycerol lipase regulates a fatty acid network that promotes cancer pathogenesis. Cell.

[78] Bertin R (1976). Glycerokinase activity and lipolysis regulation in brown adipose tissue of cold acclimated rats. Biochimie.

[79] Hammond VAJ, Desmond G. Substrate cycling between triglyceride and fatty acid in human adipocytes. Metabolism. 1987:308–13. 10.1016/0026-0495(87)90199-5 3550370

[80] Harper RD, Saggerson ED (1975). Some aspects of fatty acid oxidation in isolated fat-cell mitochondria from rat. Biochem J.

[81] Wang T, Si Y, Shirihai OS, Si H, Schultz V, Corkey RF (2010). Respiration in adipocytes is inhibited by reactive oxygen species. Obesity (Silver Spring).

[82] Wolfe RR, Klein S, Carraro F, Weber JM (1990). Role of triglyceride-fatty acid cycle in controlling fat metabolism in humans during and after exercise. Am J Physiol.

[83] Mottillo EP, Balasubramanian P, Lee YH, Weng C, Kershaw EE, Granneman JG (2014). Coupling of lipolysis and *de novo* lipogenesis in brown, beige, and white adipose tissues during chronic β3-adrenergic receptor activation. J Lipid Res.

[84] Veliova M, Ferreira CM, Benador IY, Jones AE, Mahdaviani K, Brownstein AJ, et al. Blocking mitochondrial pyruvate import in brown adipocytes induces energy wasting via lipid cycling. EMBO Rep. 2020:e49634. 10.15252/embr.201949634. 10.15252/embr.201949634 PMC7726774 33275313

[85] Nolan CJ, Madiraju MS, Delghingaro-Augusto V, Peyot ML, Prentki M (2006). Fatty acid signaling in the beta-cell and insulin secretion. Diabetes.

[86] Gauthier MS, Miyoshi H, Souza SC, Cacicedo JM, Saha AK, Greenberg AS (2008). AMP-activated protein kinase is activated as a consequence of lipolysis in the adipocyte: potential mechanism and physiological relevance. J Biol Chem.

[87] Faergeman NJ, Knudsen J. Role of long-chain fatty acyl-CoA esters in the regulation of metabolism and in cell signalling. Biochem J. 1997;323(Pt 1):1–12. 10.1042/bj3230001. 10.1042/bj3230001 PMC1218279 9173866

[88] Przybytkowski E, Joly E, Nolan CJ, Hardy S, Francoeur AM, Langelier Y (2007). Upregulation of cellular triacylglycerol - free fatty acid cycling by oleate is associated with long-term serum-free survival of human breast cancer cells. Biochem Cell Biol.

[89] Tordjman J, Chauvet G, Quette J, Beale EG, Forest C, Antoine B (2003). Thiazolidinediones block fatty acid release by inducing glyceroneogenesis in fat cells. J Biol Chem.

[90] Raimondo A, Rees MG, Gloyn AL (2015). Glucokinase regulatory protein: complexity at the crossroads of triglyceride and glucose metabolism. Curr Opin Lipidol.

[91] van Schaftingen E, Vandercammen A, Detheux M, Davies DR (1992). The regulatory protein of liver glucokinase. Adv Enzyme Regul.

[92] Sharma A, Oonthonpan L, Sheldon RD, Rauckhorst AJ, Zhu Z, Tompkins SC, et al. Impaired skeletal muscle mitochondrial pyruvate uptake rewires glucose metabolism to drive whole-body leanness. Elife. 2019;8. 10.7554/eLife.45873. 10.7554/eLife.45873 PMC6684275 31305240

[93] Grundlingh J, Dargan PI, El-Zanfaly M, Wood DM (2011). 2,4-dinitrophenol (DNP): a weight loss agent with significant acute toxicity and risk of death. J Med Toxicol.

[94] Duchen MR (2004). Roles of mitochondria in health and disease. Diabetes.

[95] Lowell BB, Spiegelman BM (2000). Towards a molecular understanding of adaptive thermogenesis. Nature.

[96] Periasamy M, Herrera JL, Reis FCG (2017). Skeletal muscle thermogenesis and its role in whole body energy metabolism. Diabetes Metab J.

[97] Bal NC, Maurya SK, Singh S, Wehrens XH, Periasamy M (2016). Increased reliance on muscle-based thermogenesis upon acute minimization of brown adipose tissue function. J Biol Chem.

